# Book Reviews

**Published:** 1914-08

**Authors:** 


### BOOK REVIEWS

---

Books mentioned may be inspected at and ordered through this office. So far as possible, books received in any month will be reviewed in the issue of the second month following.

---

**Progressive Medicine,** Vol. 16, No. 2, issued June 1, 1914. Edited by H. A. Hare and L. F. Appleman of Philadelphia. Quarterly, $6.00 per annum.. Published by Lea & Febiger.

This number of 460 pages includes the discussion of Hernia by Wm. B. Coley, Surgery of the Abdomen by John C. A. Gerster, Diseases of the Blood, Diathetic and Metabolic Diseases, etc., by Alfred Stengel and Ophthalmology by Edward Jackson. The general standard of excellence is maintained.

---

**Report of the Commissioner of Education,** for the year ending June 30, 1913. Published by the Department of the Interior of the U. S.

This is an interesting compilation of various statistics and includes the discussion of many problems in education along all lines. As the details regarding medical education are necessarily nearly a year behind the reports prepared by the A. M. A., we refer our readers to the next Educational number of the Journal of the A. M. A., to be expected late in August.

---

**Third Yearly Report of the Warsaw Hospital.** This report contains not only details regarding the hospital itself but interesting descriptions of cases treated. It reflects great credit on the Resident Surgeon, Dr. W. Ross Thompson, Fellow of the American College of Surgeons. This hospital is a conspicuous example of the tendency toward surgical decentralization, a tendency involving many interesting phases of medical economics, of surgical efficiency, of life saving by time saving, etc.

---

**Radium and Radiotherapy. Radium, Thorium and other Radio-Active Elements in Medicine and Surgery.** By William S. Newcomet, M. D., Professor of Roentgenology and Radiology, Temple University, Medical Department; Physician to the American Oncologic Hospital, Fellow of the College of Physicians, Philadelphia. 12mo, 315 pages, with 71 illustrations and 1 plate. Cloth, $2.25, net. Lea & Lebiger, Publishers, Philadelphia and New York, 1914.

This book devotes more than the usual amount of space to accurate descriptions of the mensuration of radio-activity and the economic chemistry and manufacture of radio-active substances. It does not neglect, however, the therapeutic and prophylactic problems involved. It is brief and clear.

**Blood Pressure in Medicine and Surgery. A Guide for Students and Practitioners.** By Edward H. Goodman, M. D., Associate in Medicine in the University of Pennsylvania. 12mo, 226 pages, illustrated. Cloth, $1.50, net. Lea & Febiger, Publishers, Philadelphia and New York, 1914.

The attention paid to the underlying physiology of circulation and the references to venous and capillary pressure deserve special attention from the reader. Our knowledge of blood pressure standards in various diseases, physiologic conditions, such as pregnancy and labor, under the influence of drugs, acutely and used habitually, as tobacco and alcohol, is codified in a simple and definite way to facilitate reference. Cardiac, arterial and renal abnormalities are not unduly emphasized but the subject is broadly treated.

**Biologic Products and How to Use Then.** Pamphlet, issued by the Abbott Alkaloidal Co. of Chicago, to accompany their price list. This contains in brief, practical form, our present knowledge regarding this range of therapeutic and prophylactic agents.

**Quarantine Procedure.** This Bulletin, No. 64 of the Public Health Service, is designed especially for the guidance of ship captains but is of professional interest, if for no other reason, for the map showing the sites of infectious diseases against which quarantine is maintained.

**Poor Richard Lactopeptine Almanack** for 1914. Published by the N. Y. Pharmacal Association of Yonkers, N. Y. This booklet contains a little of the calendar, beginning with an excellent comic chart of the Signs of the Zodiac; a good deal of humor in prose and rhyme, and not too much good advertising. If any physician has failed to receive it, it contains enough laughs to pay for the postage. It surely is disinterested in a firm to reduce the need for its own product, as laughter is said to do.

**Practical Therapeutics,** Daniel M. Hoyt, M. D., Philadelphia, 2d revised edition published by the C. V. Mosby Co., St. Louis; 426 pages, $5.00. This work includes materia medica and prescription writing and the scope of the materia medica is augmented by the inclusion of non-officinal remedies passed by the Council on Pharmacy and Chemistry of the A. M. A. The author employs neither the alphabetic arrangement nor the older attempt at formal classification of drugs according to physiologic action, but a very practical one indicated by the following chapter headings: Drugs that lessen the Excitability of the Nervous System; Drugs Used to Unload the Bowels; Drugs Used to Increase the Coagulability of the Blood; Drugs Used to Lessen Secretion or Excretion.

**Ten Sex Talks to Boys Ten Years and Older,** by I. D. Steinhardt, M. D., published by the J. B. Lippincott Co., Philadelphia. 187 pages, $1.00.

This work contains the elements of sexual anatomy, physiology and pathology, expressed in simple language and discusses the related moral, ethical and hygienic problems. As stated for the corresponding book for girls, we rather question whether children should have such literature placed directly in their hands for whether they should be instructed wholesale by lectures. Even more than adults, children are highly individualized. A very few years' difference in age means a very different viewpoint but children of the same age differ widely in their mental attitude and in their receptivity of this or other instruction. In our opinion, the book should be bought by every parent but used by him or her as a guide to direct oral instruction of children, at such times and in such ways as may seem best.

**Practical Pediatrics,** Drs. James H. McKee and Wm. H. Wells, and an Appendix on Development and its Anomalies by Dr. John Madison Taylor, all of Philadelphia. 2 volumes, 1180 pages, copiously illustrated, $12. P. Blakiston, Son & Co., Philadelphia.

This is the largest and most complete work on the subject that we have reviewed. It is so complete and so excellent, that it is scarcely necessary to enter into details, further than to explain that the Appendix is uniform with the rest of the work, occupies about 200 pages and is not, as the title might suggest, a treatise on embryology but a practical, clinical discussion of the management of childhood from the hygienic and sociologic standpoint.

---

**Handbook of Psychology and Mental Disease,** Dr. C. B. Burr, Flint, Mich., 4th edition, 235 pages, illustrated, $1.50. F. A. Davis Co., Philadelphia.

Dr. Burr is Medical Director of the Oak Grove Hospital and, from his ample experience, he has prepared this work especially for training schools for attendants and nurses, but also with a view to the needs of medical students and practitioners. It is not essentially a work on pathology, diagnosis and treatment but rather one of general comprehension and management. It deals with practical points which the attendant or nurse should know and which the physician is likely to overlook in his directions to the attendant. This limitation of scope gives the book special value. We venture to point out that, from the list of special senses, those of equilibrium and temperature have been omitted.

---

**Les Prejuges en Art Dentaire,** Dr. E. Charezieux, Paris; 120 pages, 3 francs, published by A. Maloine, Paris. The author states that he has chosen this title because persons who are no longer children do not have a brain like a planed table but possess notions, partly just, partly erroneous, in a word, they are victims of prejudices. However, the word has a broader meaning in French and can, perhaps, be best expressed by "general principles." A good idea of the scope of the book and its applicability to the needs of the physician can be obtained by a nearly literal translation of some of the chapter headings: "When the teeth are painful, it is, indeed, time to consult;" "If caries is extensive, better have recourse to extraction;" "Non-painful roots can be saved with Impunity;" "Lancing the gums is an excellent means of conserving the denture;" "Principles relating to procedures of local anaesthesia;" "To extract a tooth, one must pull hard and work quickly;" "Never extract when there is an abscess;" "Putting cotton in the ears prevents dental diseases—Lesions of the Eye-tooth are the gravest;" "Conceptions regarding prosthetic apparatus." In a personal letter, the author states that the book has been written especially to give the medical profession an intelligent point of view of the possibilities of dentistry. We think that the chapter headings put the dental problems very much as they occur to the physician, either as an advisor or as an emergency practitioner of dentistry. Each one is rationally discussed.

